# Identification of a novel peptide that blocks basic fibroblast growth factor-mediated cell proliferation

**DOI:** 10.18632/oncotarget.1312

**Published:** 2013-10-01

**Authors:** Xiaoping Wu, Huixian Huang, Cong Wang, Shaoqiang Lin, Yadong Huang, Yi Wang, Guang Liang, Qiuxia Yan, Jian Xiao, Jianzhang Wu, Yongguang Yang, Xiaokun Li

**Affiliations:** ^1^ School of Pharmaceutical Science, Key Laboratory of Biotechnology and Pharmaceutical Engineering of Zhejiang Province, Wenzhou Medical College, Wenzhou, PR China; ^2^ Institute of Tissue Transplantation and Immunology, Jinan University, Guangzhou, PR China; ^3^ National Engineering Research Center for Gene Medicine, Jinan University, Guangzhou, PR China and; ^4^ Columbia Center for Translational Immunology, Columbia University Medical Center, New York, USA

**Keywords:** bFGF, phage display, proliferation, cancer therapy

## Abstract

Basic fibroblast growth factor (bFGF) has been implicated in tumor growth via interactions with its receptors (FGFRs) on the cell surface and therefore, bFGF/FGFRs are considered essential targets for cancer therapy. Herein, a consensus heptapeptide (LSPPRYP) was identified for the first time from a phage display heptapeptide library after three sequential rounds of biopanning against FGFR-expressing cells with competitive displacement of phage by bFGF, followed by subtraction of non-specific binding by FGFR-deficient cells. Phage bearing LSPPRYP showed high levels of binding to Balb/c 3T3 cells expressing high-affinity bFGF-binding FGFR (bFGFR), but not to the cells that do not express bFGFR (Cos-7), or express a very low affinity bFGFR (HaCat). The selected-phage-derived peptide synthesized by solid phase method using a rapid and practical Fmoc strategy was found to specifically compete with bFGF for binding to its receptors, inhibit bFGF-stimulated cell proliferation by inducing cell cycle arrest, and block bFGF-induced activation of Erk1 and Erk2 kinase in B16-F10 melanoma cells. Importantly, treatment of melanoma-bearing mice with the synthetic peptide significantly suppressed tumor growth. The results demonstrate a strong anticancer activity of the isolated bFGFR-binding peptide (and its future derivatives), which may have great potential for cancer therapy.

## INTRODUCTION

Basic fibroblast growth factor (bFGF) plays essential roles in both physiological and pathological processes by interacting with specific receptors on the cell surface [1, 2]. Because overexpression of bFGF and/or its receptors is commonly detected in tumors, the development of antagonists to bFGF and its receptors has been considered as a potential strategy for cancer therapy [3, 4].

Phage display technology has been used to identify desirable peptides and is an important tool for drug discovery. We have successfully applied the technique to identify a high-affinity bFGF-binding peptide with strong inhibitory activity against bFGF-induced cell proliferation and angiogenesis [5]. Herein, the goal was to isolate a FGF receptor (FGFR)-binding peptide with this technique. The phage display library used consisted of phages bearing random heptapeptides fused to the N-terminus of the coat protein pIII of the M13 Phage. Using FGFR-deficient Cos-7 cells as the subtractive cells, three consecutive rounds of panning with the library against Balb/c 3T3 cells expressing exuberant FGF receptors were performed to identify a binding peptide sequence (LSPPRYP), by extrapolating from the corresponding DNA sequences of 5 positive phage clones eluted from the cells by bFGF. The phage bearing LSPPRYP was evaluated for its cell-binding specificity. Moreover, because bFGF greatly contributes to melanoma growth and progress [6, 7], the synthetic peptide consisting of this sequence and a spacer sequence GGGS was further tested for antitumor activity using B16-F10 murine melanoma cells for *in vitro* experiments, and introduced into C57BL/6 mice for *in vivo* experiments. The results demonstrated that the identified synthetic peptide could reverse the effects of bFGF on cell proliferation, cell cycle progression, Erk1/Erk2 activation of melanoma cells, and significantly inhibit tumor growth in mice.

## RESULTS

### Isolation of phages binding to bFGF receptors

**Figure 1:**
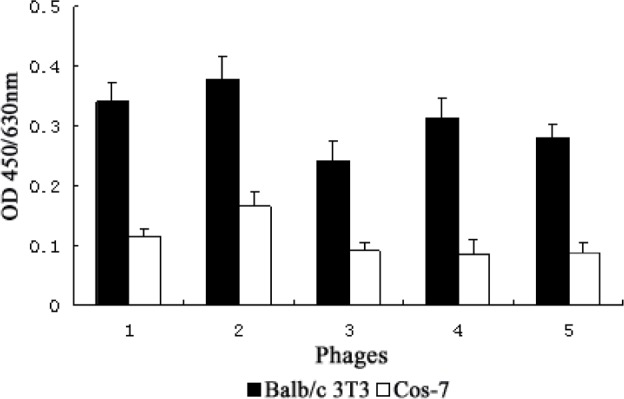
Specific binding of the positive phage clones to bFGF receptors

Specific phages capable of binding to bFGF receptors were selected by three rounds of biopanning against positive cells expressing high-affinity bFGF receptors on the cell surface. In order to diminish the background of screening, bound phages were specifically eluted with bFGF and subtractive panning was carried out against cells that were deficient in bFGF receptors. In the first round, a lower concentration of PBST (0.05%) was applied to wash for higher eluate titers. In order to enrich highly specific and affinity phages, nonspecifically binding phages were absorbed by subtractive cells before screening, and the concentration of PBST was then increased to 0.1% from the second round. In the last round of panning, low affinity phages eluted within 1 h were discarded, and the phages further eluted with bFGF for an additional 1 h were analyzed by ELISA to identify high-affinity bFGF receptor-binding clones. Phage clones that exhibited a binding affinity (i.e, OD value) to Balb/c 3T3 2-fold greater than observed for Cos-7 cells were considered positive. As shown in Fig. 1, we identified 5 positive clones from a total of 13 phage clones.

The binding affinities of 5 positive phage clones to Balb/c 3T3 cells and Cos-7 cells were detected by ELISA assay. Data presented are the mean OD values (±SDs) of triplicate samples.

### Sequence analysis and property prediction of positive phages

Total DNA of the positive phages was isolated and sequenced using −96gⅢ primers. The amino acid sequences of the peptides displayed on the corresponding phages were deduced from the DNA sequences and Bioedit and ProtParam programs were applied to analyze the sequences and predict the peptide properties. As shown in Table 1, 5 clones shared consensus sequences (LSPPRYP). Comparison of the amino acid sequences of the heptapeptide (P9) with that of bFGF revealed that the P9 contained 6 amino acids identical to the adjacent amino acids (L3, S9, P13, P14, R120, Y124) of the 3D structure of bFGF, which are located within the motifs (P13~K18 and R120~K125), which are involved in receptor binding and mitogenic activity of bFGF. Furthermore, similar to bFGF, P9 also carried positive charges under physiological conditions, suggesting that electrostatic interaction might also be involved in their binding to FGF receptors.

**Table 1:** Properties of peptides displayed by positive phages

Heptapeptide	Clone	Sequence	Similarity	Theoretical pI^a^	GRAVY^b^
P9	1~5	LSPPRYP	0.0479452	8.75	−1.086

a pI, Isoelectric Point.

b GRAVY, Grand Average of Hydropathicity.

### Specificity of selected phage clone for binding cells

**Figure 2:**
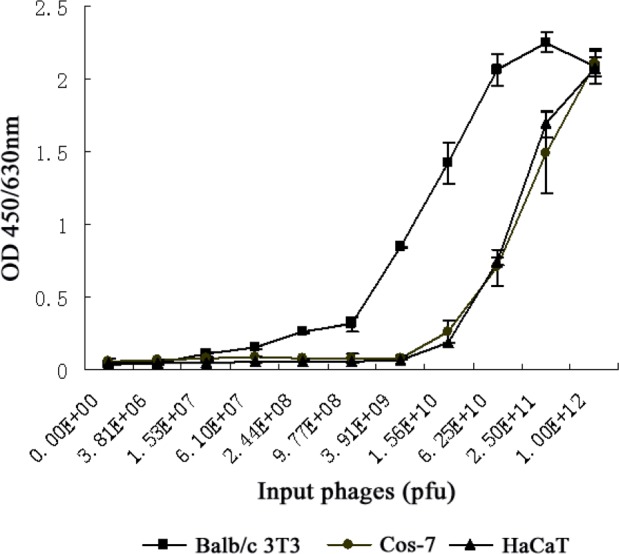
Comparison of binding affinity of LSPPRYP phage for different cell lines

It has been shown that Balb/c 3T3 cells express high-affinity bFGF receptors (e.g., FGFR1c and FGFR2c) on the cell surface, while HaCat cells exclusively express a specific isoform of FGFR2 (also known as FGFR2b or KGFR) with a very low affinity to bFGF [8, 9]. To assess the binding specificity of the selected phage clone, we compared the ability of the phages to bind Balb/c 3T3, HaCat and FGFR-deficient Cos-7 cells [10, 11]. As shown in Figure 2, the affinity of the phage clone LSPPRYP to Balb/c 3T3 cells was markedly stronger than to HaCaT and Cos-7 cells. The Kd value for Balb/c 3T3 cells was between 3.91×10^9^ pfu and 1.56×10^10^ pfu, which is approximately 16 times less than the Kd value (between 6.25×10^10^ pfu and 2.50×10^11^ pfu) for HaCaT and Cos-7 cells (Fig. 2). The results revealed that the LSPPRYP phage exhibits greater binding to the cells expressing high-affinity bFGF receptors than to the cells with low affinity bFGF receptors or without bFGF receptors.

Phages were first incubated with Balb/c 3T3, HaCat, and Cos-7 cells respectively, and then washed with PBST. The quantity of the phages binding to the cells was detected with anti-M13 mAb. Data presented are the mean OD value (±SDs) of triplicates.

### Specificity of the synthetic peptide for bFGF binding to its receptors

**Figure 3:**
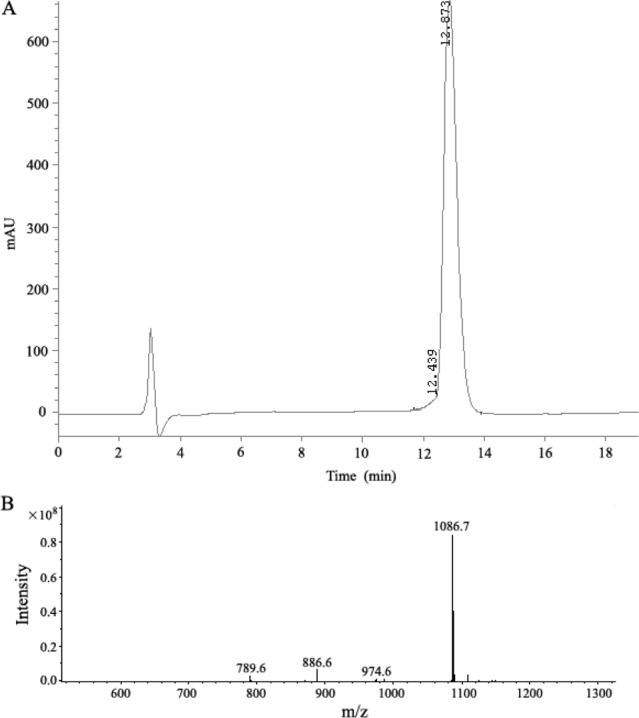
Analysis of the synthetic P9 peptides

In a phage display heptapeptide library, the C-terminus of the random peptide is fused to the phage via a short spacer (GGGS), having no free negatively charged carboxylate. Therefore, a spacer sequence (GGGS) was added to the C-terminus and the C-terminal carboxylate was amidated to block the negative charge when synthesizing the peptide corresponding to the selected sequence (LSPPRYP). The selected-phage-derived peptide was synthesized on solid phase using a rapid and practical Fmoc strategy. The crude compound was subjected to RP-HPLC purification. As shown in Fig. 3, high purity grade (98%) was obtained after purification and the synthetic peptide was characterized for its identity by mass spectrometry analysis.

(a) HPLC chromatogram at 214 nm. (b) electrospray ionization mass spectrum of the synthetic peptide.

**Figure 4:**
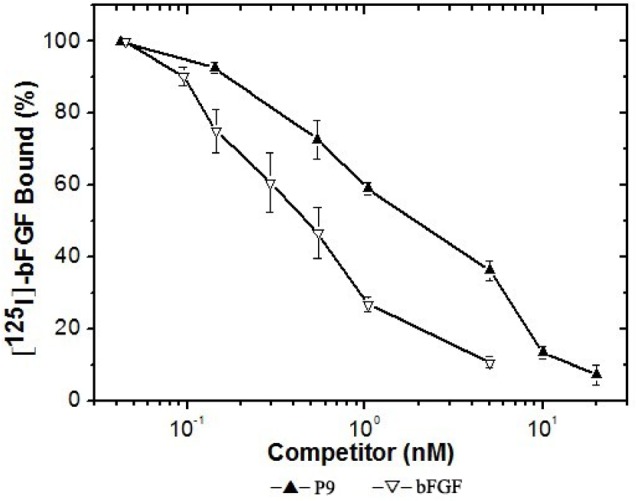
Specific binding of 125I -bFGF to Balb/c 3T3 cells competed by increasing concentrations of bFGF or the synthetic P9 peptides

To characterize the effect of the synthesized P9 peptides (LSPPRYPGGGS) on bFGF binding to its receptors, P9 peptides were compared to bFGF for their ability to compete directly for ^125^I -bFGF binding on Balb/c 3T3 cells expressing exuberant bFGF receptors. As shown in Fig. 4, P9 is highly efficient in specific competing for ^125^I-bFGF binding to its receptors, in which about 2.5 nM of P9 was able to displace 50% of ^125^I-bFGF. Scatchard analysis of ^125^I-bFGF binding on Balb/c 3T3 revealed a receptor population consisting of approximately 9,600 sites/cell with a dissociation constant (K_D_) of 100 pM. These affinity and capacity values are within the range of values previously published for the high affinity bFGF receptors (K_D_ = 10 - 200 pM, 0.2-10 ×10^4^ sites/cell). In the presence of P9 peptides, specific high affinity binding of ^125^I-bFGF to Balb/c 3T3 cells decreased significantly with a K_D_ of 500 pM and a receptor population of about 27,000 sites/cell. These results indicate that P9 peptides are able to block the binding of bFGF to its receptors, and may have the potential to antagonize bFGF bioactivity.

Binding assays were performed as described in Materials and Methods. Values shown are the mean value (±SDs) of triplicates.

### Effect of the synthetic peptide on bFGF-stimulated proliferation of melanoma cells

**Figure 5:**
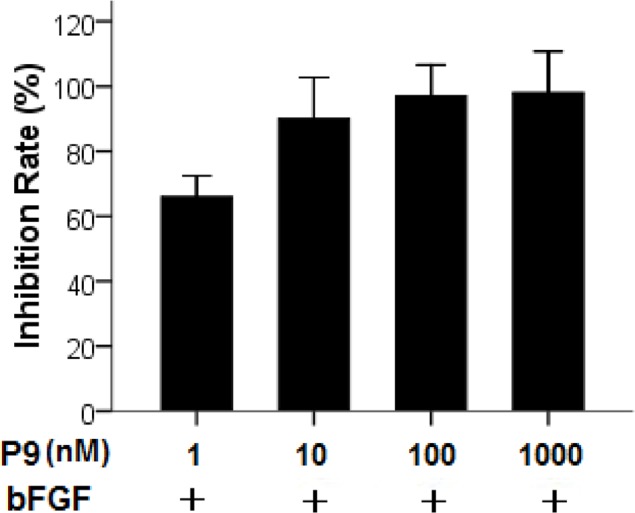
Inhibition of bFGF-stimulated proliferation of B16-F10 cells by the synthetic P9 peptides

We next determined the efficacy of the synthetic P9 peptides in blocking bFGF-stimulated tumor cell proliferation. B16-F10 murine melanoma cells with high levels of FGFR expression [12, 13] were incubated with 30 ng/mL bFGF alone or along with various concentrations of the P9 peptides. As shown in Fig. 5, the synthetic peptides inhibited B16-F10 cell proliferation in a dose-dependent manner and nearly 90% of inhibition was achieved at 10 nM.

Starved cells were treated with 30 ng/ml bFGF alone, or 30 ng/ml bFGF plus P9 at the indicated concentrations. Cell proliferation was determined 48 h after treatment. Data are presented as the mean (±SDs) of 3 independent experiments performed in triplicate.

### Effect of the synthetic peptides on bFGF-induced cell cycle progression

**Figure 6:**
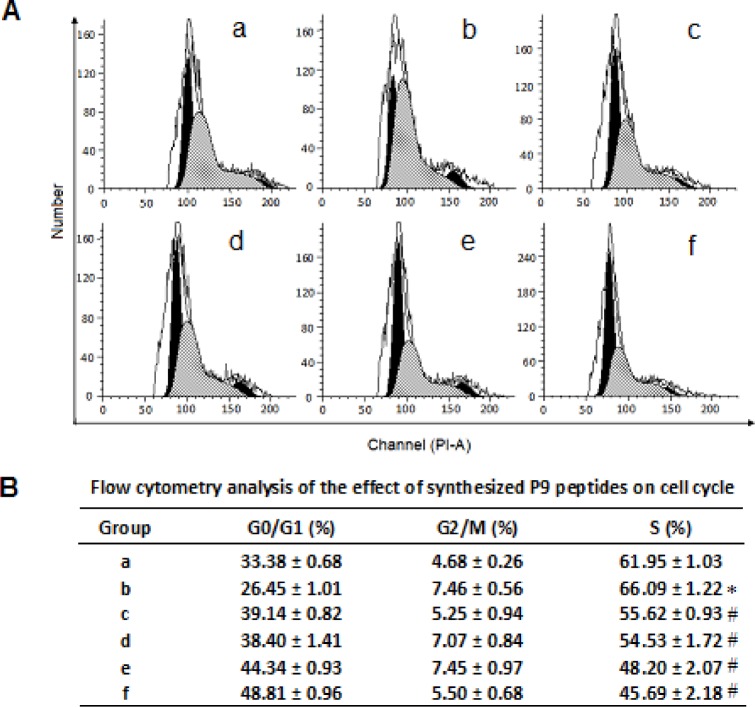
Flow cytometry analysis of the effect of the synthetic P9 peptides on cell cycle distribution of bFGF-stimulated cells

The effect of the synthetic peptides on cell cycle progression induced by bFGF was determined by flow cytometry analysis. As shown in Fig. 6, treatment with bFGF significantly increased the percentage of S phase cells, and decreased the ratio of G0/G1 phase cells. In contrast, cells treated with bFGF plus the synthetic peptides had a higher G0/G1-phase population and lower S-phase population than those treated with bFGF alone, suggesting that the synthetic peptides reversed the S-phase increase induced by bFGF, and arrested the cells at the G0/G1 phase.

Panel A, (a) Control cells without treatment of bFGF or P9. (c-f) B16-F10 cells were starved for 24 h and then treated with (b) 30 ng/ml bFGF, (c) 30 ng/ml bFGF plus 1 nM P9, (d) 30 ng/ml bFGF plus 10 nM P9, (e) 30 ng/ml bFGF plus 100 nM P9, or (f) 30 ng/ml bFGF plus 1000 nM P9 for 48 h. Cells were then fixed in 70% ice-cold ethanol, washed with PBS, stained with PI, and subjected to flow cytometry analysis. Data shown are the representative pictures of 3 independent experiments. Panel B, cell cycle distribution of the control and treated cells. Data are presented as the mean (±SDs) of 3 independent experiments. * *P*< 0.01 *vs* control group; # *P*< 0.01 *vs* bFGF group.

### Effect of the synthetic peptides on bFGF-induced MAP kinase activation

**Figure 7:**
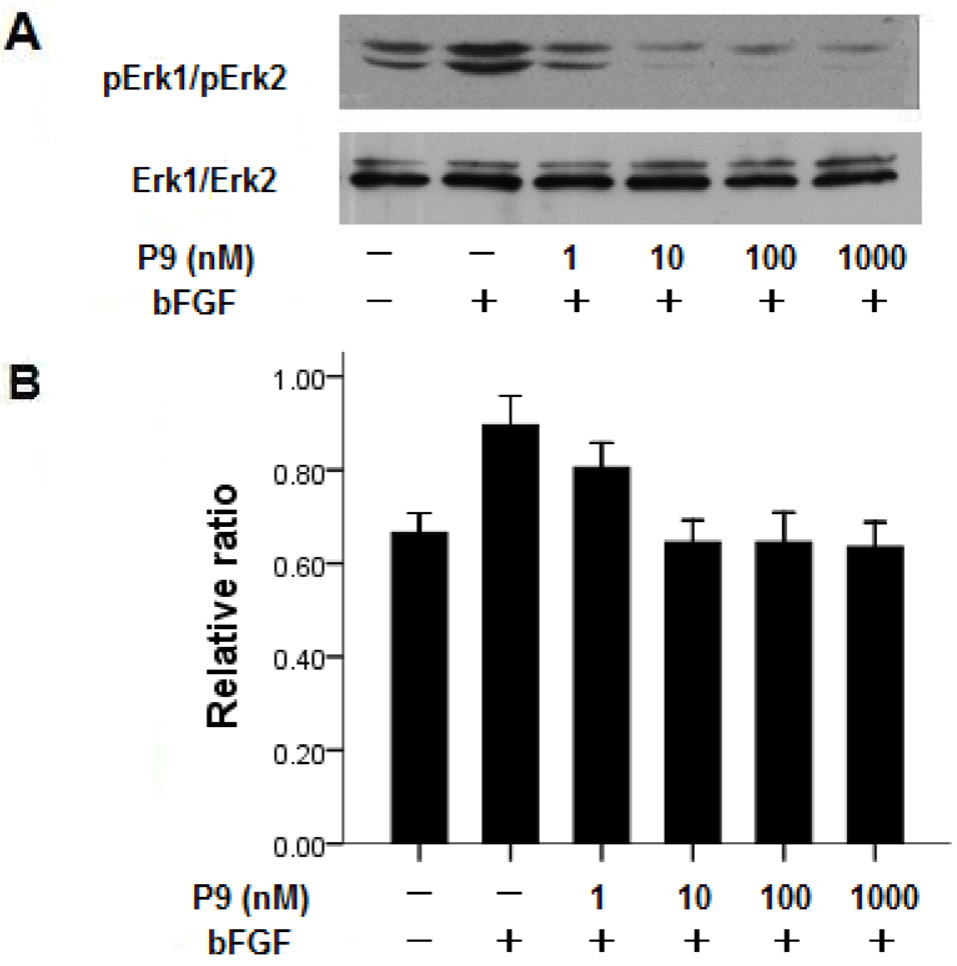
Synthetic P9 peptides inhibit bFGF-induced MAP kinase activation

The effect of the synthetic P9 peptide on bFGF signal transduction was determined by measuring its capacity to inhibit the activation of two MAPKs (Erk1 and Erk2) in B16-Fl0 cells after stimulation by bFGF. Pretreatment of B16-Fl0 cells with the synthetic peptides at a concentration of 10 nM or higher for 5 min prior to bFGF stimulation completely suppressed bFGF-induced Erk1 and Erk2 phosphorylation (Fig. 7, lanes 3-6). The levels of Erk1/Erk2 activation in the cells treated with bFGF and P9 peptides at a concentration of 10 nM or higher were significantly lower than those in the cells treated with bGFG alone, and comparable to the untreated control B16-Fl0 cells.

Panel A. Before stimulation with 30 ng/ml bFGF for 20 min, starved B16-F10 cells were pretreated with P9 at the indicated concentrations for 5 min. Cell lysates were resolved with 10% SDS-PAGE, transferred to PVDF membrane, and blotted with anti-pErk1/pErk2 mAb. Data shown are the representative pictures of 3 independent experiments. Panel B. Density ratios of pErk1/pErk2 to Erk1/Erk2. Data are presented as the mean (±SDs) of 3 independent experiments.

### The synthetic peptides suppressed tumor growth *in vivo*

**Figure 8:**
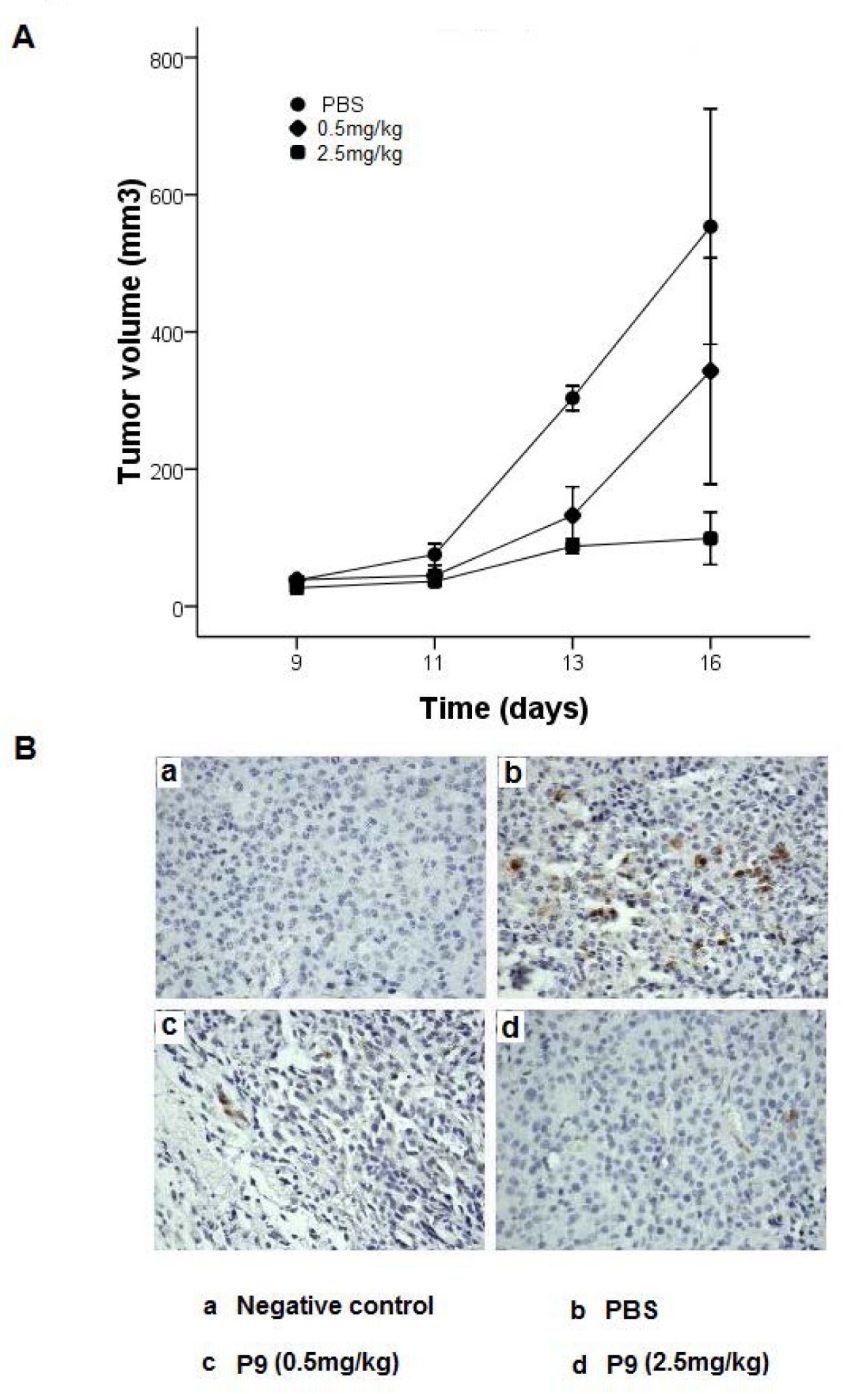
Synthetic P9 peptides inhibit the growth of the murine melanoma B16-F10 cells in mice

We further investigated the *in vivo* antitumor efficacy of the synthetic peptides in a mouse model of melanoma. Tumor was established by subcutaneously implanting the C57BL/6-derived B16-Fl0 cells into C57BL/6 mice. The synthetic peptides were administrated at dose of 0.5 mg/kg or 2.5 mg/kg on alternate days starting at day 7, and the growth of the tumors was assessed for 9 days. Treatment with the synthetic peptides resulted in a significant reduction in tumor volume compared to that observed as a result of PBS treatment (*P*<0.01 *vs* PBS group). A dose-dependent inhibition of tumor growth of 38% and 82% was observed on day 9 for animals treated with the synthetic peptides at a dose of 0.5 mg/kg and 2.5 mg/kg, respectively (Fig. 8A). All mice were sacrificed at day 9, one day after the last injection of the P9 peptides (16 days after tumor injection, and 9 days after first administration of the synthetic peptides). Immunostaining with anti-phospho-Erk1/Erk2 (pErk1/pErk2) antibody showed a dose-dependent inhibition of Erk1/Erk2 activation. The numbers of pErk1/pErk2-positive cells were markedly reduced in tumors from mice treated with P9 peptides compared to tumors in control animals (Fig. 8B). The data showed a potent inhibitory effect of the P9 peptides on tumor growth *in vivo*, which was associated with significant inhibition of Erk1/Erk2 activation in tumor cells.

(A) B16-F10 cells were injected subcutaneously into female C57BL/6 mice. When tumors were visible on all mice on day 7, treated mice were intraperitoneally injected with P9 on alternate days at 0.5, or 2.5 mg/kg, whereas control mice were injected with PBS (n=10 mice for each group). (B) P9 inhibits Erk1/Erk2 activation in tumors *in vivo*. On the next day after last injection, the B16-F10 tumors were extracted, fixed, and embedded in paraffin. Tissue sections were immunostaining for phospho-Erk1/2 as described in the Material and Methods. Meanwhile, negative control was incubated with PBS instead of monoclonal anti-phospho-Erk1/2. Negative control (Panel a), PBS (Panel b), 0.5 mg/kg (Panel c), and 2.5 mg/kg (Panel d). Data are representative of 2 independent experiments.

## DISCUSSION

Phage display technology provides an efficient tool to identify the desirable sequences binding specifically to targets, and is widely used in diagnostic and therapeutic protein developments in recent years [14-16]. FGFRs have been shown to act as driving oncogene in certain cancers to maintain the malignant properties of tumor cells via a cell autonomous manner [17]. In this study herein, we developed a biopanning strategy using phage display technology for identifying bFGF receptor-binding peptides. The selected phage clone exhibits a remarkable binding affinity to Balb/c 3T3 cells that express high-affinity bFGF receptors, while binding poorly to cells that do not express bFGF receptors (Cos-7), or express low affinity bFGF receptors. Furthermore, the identified peptide specifically competed for bFGF binding to its receptors, and was able to inhibit bFGF-stimulated cancer proliferation *in vitro* and suppress tumor growth *in vivo*.

There is a growing body of evidence that bFGF promotes cancer cell proliferation through the activation of the Ras/Raf/MEK/ERK pathway [18-21]. Cell proliferation is regulated during the G0/G1 phase in the cell cycle. CDK4 and CDK6 interacting with the cyclin D family of proteins drive G0/G1 phase into the DNA synthesizing S-phase [22]. ERK activity plays a pivotal role in cell proliferation via regulation of cyclin D1 expression during mid-G1 and driving cells past the G1-restriction point [23, 24]. In order to explore the potential of the isolated bFGF receptor-binding peptide P9 in cancer treatment, we investigated the effects of P9 on bFGF-induced proliferation, cell cycle distribution, and MAKP signal transduction in B16-F10 cells. Our results showed that P9 inhibited the proliferation of B16-F10 cells stimulated by bFGF in a dose-dependent manner. In accordance with the inhibitory effect of P9 on cell proliferation, cell cycle analysis showed that P9 arrested bFGF-stimulated cells at the G0/G1 phase, and the MAP kinase activation assay revealed that P9 reduced the levels of Erk1 and Erk2 phosphorylation in B16-F10 cells induced by bFGF. Downregulation of Erk1 and Erk2 activation may contribute to more G0/G1 cells in P9-treated cells than in the untreated control cells via restriction of cyclin D1 expression during mid-G1 and inhibition of cells passing the G1-restriction point. In a small animal model system, P9 significantly suppressed tumor growth and blocked Erk1/Erk2 activation in the tumors. The synthetic peptide attenuates the mitogenic activity of bFGF possibly via interfering with the interaction between bFGF and its receptors.

As bFGF/FGFRs have been considered as a target for cancer therapy, efforts have been made to develop the antagonists targeting bFGF/FGFRs including antibodies and small molecule tyrosine kinase inhibitors. When compared to antibodies, small peptides may have several advantages for therapy. First, the peptides are easy to synthesize and modify according to their pharmacokinetic requirements [25]. Second, they are small and easily penetrate through the tissue [26]. Third and most importantly, they have low immunogenicity [25, 27]. There are also disadvantages for utilizing small peptides [27]. One disadvantage is their rapid proteolysis by endogenous peptidases and proteases. There are several ways to overcome this obstacle including the usage of D-amino acids, and substitution of the peptide bonds. Another disadvantage for small peptides is the loss of binding affinity when conjugated to other therapeutic molecules, which could be addressed by keeping the conjugated molecules apart from the receptor-binding sequence. Compared to chemical agents such as tyrosine kinase inhibitors which are not tumor specific and bear the risk of severe side effects [28], P9 has potential for high selectivity because it targets interactions between bFGF and its receptors, and may be safer to use from a clinical point of view.

In summary, our results demonstrate that the isolated bFGF receptor-binding peptide P9 provides an effective bFGF/FGFR antagonist, and may have potential application for the treatment of proliferative disorders, including a variety of cancers with upregulation of bFGF/FGFRs.

## MATERIALS AND METHODS

### Reagents and cell lines

Ph.D.-7™ Phage Display Peptide Library Kit and *Escherichia coli* ER2738 were purchased from New England Biolabs Inc. (Beverly, MA, USA). Horseradish peroxidase (HRP)-anti-M13 mAb was purchased from Amersham Pharmacia Biotech (Uppsala, Sweden). Phospho-Erk1/2 (pErk1/pErk2) rabbit mAb, Erk1/2 (Erk1/Erk2) rabbit mAb and goat anti-rabbit IgG conjugated with horseradish peroxidase antibody were purchased from Cell Signaling Technology (Danvers, MA, USA). Anti-rat immunoglobulin sensitive S-P detection kit (UltraSensitive™ S-P) was purchased from Maixin Limited Corporation (Fuzhou, Fujian, China). Balb/c 3T3, HaCat, Cos-7, and B16-F10 cells were maintained in Dulbecco's modified Eagle's medium (DMEM) (Invitrogen Corporation, Carlsbad, CA, USA) with 10% fetal bovine serum (FBS).

### Biopanning of a heptapeptide phage display library

Balb/c 3T3 cells grown to 90 % confluence in 25 cm^2^ tissue culture flask were washed 2 times and blocked with DMEM-2% dry milk for 2 h at 37°C, 5% CO_2_. After the cells were washed 5 times with 0.05% Tween-20 in PBS (0.05% PBST), the heptapeptide phage library containing 2 × 10^11^ pfu diluted in 2 ml DMEM was added and agitated gently at room temperature for 1 h. The cell-bound phages were washed 10 times with 0.05 % PBST, then eluted with 555 nM bFGF with gentle agitation at room temperature for 2 h. The eluate was then amplified, purified and titered for the second round of panning. The phages containing 2 × 10^11^ pfu obtained from the first round of panning were diluted in 2 ml DMEM-2% dry milk, added to 25 cm^2^ flask with a 90 % confluency of Cos-7 cells, and agitated gently at room temperature for 1 h. The supernatant was collected and added to the flask cultured with Balb/c 3T3 cells. Steps were repeated similar to the first round of panning except that the concentration of Tween-20 in PBS used for washing was increased from 0.05% to 0.1%. For the last round of selection, low affinity cell-bound phages were first eluted with bFGF for 1 h, discarded, and high-affinity cell-bound phages were then eluted with bFGF for an additional 1 h. The phage clones obtained from the eluate were subsequently subjected to ELISA analysis.

### ELISA assay for positive phages

Balb/c 3T3 cells and Cos-7 cells (negative control) were plated at a density of 1 × 10^4^ cells per well in a 96-well plate and incubated at 37°C, 5% CO_2_ for 24 h. After the plates were blocked with PBS-2% dry milk (PBSM) at 37°C for 1 h and washed 3 times with 0.05% PBST, phage clones (~10^10^ pfu/well) serially diluted 4 times were added and incubated with gentle agitation at room temperature for 1 h. PBS was used as the blank control. The plates were then washed 3 times with 0.05% PBST before 200 μl of HRP-anti-M13 mAb (1:5000) was added to each well, and incubated with gentle agitation at room temperature for 1 h. After 3 washes with 0.05 % PBST, 50 μl of freshly-made 3,3',5,5'-tetramethylbenzidine (TMB) solution was added to each well and kept at room temperature in the dark for 20 min. The reaction was terminated by adding 50 μl of 2 M H_2_SO_4_ to each well. Absorbance was measured at 450 nm with reference at 630 nm.

### Evaluation of cell-binding specificity of selected phage clone

Balb/c 3T3, HaCaT and Cos-7 were grown to confluence in 96-well plates. After the plates were blocked with PBSM at 37°C, 5% CO_2_ for 1 h and washed 3 times with 0.05% PBST, phage clones (~10^12^ pfu/well) serially diluted 4 times were added and incubated at room temperature for 1 h with gentle agitation. PBS was used as the blank control. The cells were washed briefly with PBST before incubation with HRP-anti-M13 mAb (1: 5000) at room temperature for 1 h. After 3 washes with PBST, freshly-made TMB solution was added to react at room temperature in the dark for 20 min. The reaction was terminated and the absorbance was measured in the manner as described earlier.

### DNA sequencing and peptide synthesis

DNA sequences of the positive phage clones were determined using an automated DNA sequencer at Invitrogen Company (Shanghai, China), and analyzed with Bioedit and ProtParam programs (http://au.expasy.org/tools/protparam.html).

In order to mimic the structure of peptide displayed on the phage, a spacer sequence Gly-Gly-Gly-Ser was added to the C-terminus, and the C- terminal carboxylate was amidated for blocking the negative charge when the peptide corresponding to the selected sequence was synthesized on a Liberty Microwave Peptide Synthesizer (CEM Corp., Matthews, NC) by solid phase method following the Fmoc chemistry protocols. Briefly, the first amino acid derivative Fmoc-Ser(tBu)-OH was loaded on the amide resin, and the peptide chain was elongated by sequential coupling and Fmoc deprotection of Fmoc-Gly-OH, Fmoc-Gly-OH, Fmoc-Gly-OH, Fmoc-Pro-OH, Fmoc-Tyr(tBu)-OH, Fmoc-Arg(Mtr)-OH, Fmoc-Pro-OH, Fmoc-Pro-OH, Fmoc-Ser(tBu)-OH, and Fmoc-Leu-OH. The peptide was then cleaved from the solid support and simultaneously removed of all protecting groups. The crude product was isolated by precipitation, dried in vacuo, and purified by RP-HPLC with a C18 HPLC column. A gradient of 30–100% acetonitrile containing 0.1% trifluoroacetic acid was applied to elute the column with continuous measurement of the absorbance at 214 nm. The synthetic product was further confirmed by mass spectrometry analysis.

### Receptor binding assay

The recombinant bFGF was iodinated by the modified chloramine-T method. Briefly, bFGF (5 μg/50 μ1 in 0.5 M phosphate buffer, pH 7.4) was incubated with chloramine-T (20 μg/10 μ1 of phosphate buffer) and ^125^I-labeled sodium (1.12 mCi/10 μ1) at room temperature for 55 sec. The reaction was immediately terminated by the addition of sodium metabisulflte (250 μg/200 μl) and 1% potassium iodide (100 μl), and the mixture was applied to a Sephadex G-50 column (1×20 cm) preequilibrated in 50 mM phosphate buffer containing 0.1% BSA to separate ^125^I-labeled bFGF from free ^125^I.

Confluent Balb/c 3T3 cells (0.5×10^6^/well) were incubated with binding buffer (DMEM, 20mM HEPES, pH7.4) containing ^125^I-bFGF (50000 cpm) and different concentrations of unlabeled ligand (bFGF or the synthetic peptide) for 2.5 h at 4°C. For nonspecific binding, cells were incubated with ^125^I-bFGF (50000 cpm) and an excess of unlabeled bFGF (200 nM). The cells were washed with (3 × 1 ml) cold PBS (50 mM, PH7.4), and lysed with 1% Triton X-100 (500 μl). The radioactivity of the extracts was measured in a γ counter. Specific binding was determined as the difference between total and nonspecific binding, and data were analyzed by Scatchard analysis.

### Cell proliferation assay

Cells were seeded in 96-well plates (5 × 10^3^ cells/well) in DMEM containing 10% FBS. After incubation at 37°C for 24 h, the culture medium was replaced by DMEM containing 0.4% FBS to starve the cells for 24 h. The medium was then replaced by DMEM containing 30 ng/ml bFGF, or 30 ng/mL bFGF plus peptide at various concentrations. After culturing for 48 h, the number of viable cells was determined by the methylthiazoletetrazolium (MTT) method as previously described [5].

### Flow cytometric analysis of cell cycle

Cells were starved for 24 h, and treated with 30 ng/ml bFGF or 30 ng/mL bFGF plus serially-diluted peptides for 48 h. After fixing in 70% ice-cold ethanol for 30 min at 4 °C and washing with PBS for 3 times, cells were stained with propidium iodide (PI) in the dark at room temperature for 30 min. The ModFit DNA analysis program was applied to analyze the percentages of cells at various phases of the cell cycle.

### MAP kinase activation assay

Starved cells were pretreated with serially-diluted peptides for 5 min before 20 min of stimulation with bFGF (30 ng/ml). Cells were lysed in SDS-PAGE loading buffer after being washed with cold PBS. Cell lysates were separated by 10% SDS–PAGE, then transferred to PVDF membranes, and immunoblotted, respectively, with anti-pErk1/pErk2 mAb and anti-Erk1/Erk2 mAb followed by a goat anti-rabbit IgG, HRP-linked antibody. The blots were visualized with the ECL detection system.

### *In vivo* antitumor studies

B16-F10 murine melanoma cells were harvested, and injected subcutaneously into the flanks (5 × 10^5^ cells) of 7-week old female C57BL/6 mice. When tumors were visible on all mice on day 7, treated mice were intraperitoneally injected with the synthetic peptides on alternate days for a total of 5 injections at a dosage of 0.5, or 2.5 mg/kg, whereas control mice were injected with PBS. The tumor volumes were determined by measuring length (l) and width (w) and calculating volume (V = lw^2^/2) as described elsewhere [29]. On the next day after the last injection, mice were sacrificed and the tumors were extracted and fixed in 10% neutral buffered formalin and embedded in paraffin. Tissue sections cut from the paraffin blocks were deparaffinized and processed to microwave antigen retrieval before being subjected to stain with a monoclonal rabbit anti-phospho-Erk1/2 at a dilution of 1: 200 overnight at 4°C. Meanwhile, tissue sections incubated with PBS instead of monoclonal anti-phospho-Erk1/2 were used as the negative control. Visualization of the antigen-antibody reaction was performed using an UltraSensitive™ S-P (Rabbit) kit according to the manufacturer's suggestions.

### Statistical analysis

Statistical analysis was carried out with SPSS version 13.0. One-way ANOVA analysis was applied to determine the statistical differences between groups. Data were presented as mean ± SDs. Statistical significance was accepted at *P*<0.05.
